# IL-21R Signaling Suppresses IL-17^+^ Gamma Delta T Cell Responses and Production of IL-17 Related Cytokines in the Lung at Steady State and After Influenza A Virus Infection

**DOI:** 10.1371/journal.pone.0120169

**Published:** 2015-04-07

**Authors:** Emily K. Moser, Jie Sun, Taeg S. Kim, Thomas J. Braciale

**Affiliations:** 1 The Beirne B. Carter Center for Immunology Research, The University of Virginia, Charlottesville, Virginia, United States of America; 2 Department of Pharmacology, The University of Virginia, Charlottesville, Virginia, United States of America; 3 Herman B. Wells Center for Pediatrics, The University of Indiana, Indianapolis, Indiana, United States of America; 4 Department of Pathology, The University of Virginia, Charlottesville, Virginia, United States of America; McGill University, CANADA

## Abstract

Influenza A virus (IAV) infection of the respiratory tract elicits a robust immune response, which is required for efficient virus clearance but at the same time can contribute to lung damage and enhanced morbidity. IL-21 is a member of the type I cytokine family and has many different immune-modulatory functions during acute and chronic virus infections, although its role in IAV infection has not been fully evaluated. In this report we evaluated the contributions of IL-21/IL-21 receptor (IL-21R) signaling to host defense in a mouse model of primary IAV infection using IL-21R knock out (KO) mice. We found that lack of IL-21R signaling had no significant impact on virus clearance, adaptive T cell responses, or myeloid cell accumulations in the respiratory tract. However, a subset of inflammatory cytokines were elevated in the bronchoalveolar lavage fluid of IL-21R KO mice, including IL-17. Although there was only a small increase in Th17 cells in the lungs of IL-21R KO mice, we observed a dramatic increase in gamma delta (γδ) T cells capable of producing IL-17 both after IAV infection and at steady state in the respiratory tract. Finally, we found that IL-21R signaling suppressed the accumulation of IL-17^+^ γδ T cells in the respiratory tract intrinsically. Thus, our study reveals a previously unrecognized role of IL-21R signaling in regulating IL-17 production by γδ T cells.

## Introduction

Influenza A Virus (IAV) infection of the respiratory tract triggers robust and complex immune responses which are critical to achieve virus clearance, but also can contribute to excess lung inflammation/injury and disease development. B-cell antibody production and antiviral CD8^+^ T cell responses are essential for virus clearance, since elimination of either one of these components severely impairs host elimination of virus[1,2]. In addition to important functions in virus clearance, CD8^+^ T cells also can serve as an important contributor to the development of excessive inflammation and acute lung injury after IAV infection. Therefore, disruption of factors regulating IAV-specific B cell antibody production and/or CD8^+^ T cell effector responses may have dramatic effects on virus control and the severity of lung inflammation and injury after infection.

IL-21 is an immunomodulatory type-I family cytokine produced mainly by CD4^+^ T helper cells such as Th17 and Tfh cells, and IL-21 shows structural similarity to IL-2, IL-4, and IL-15 proteins. IL-21 binds to and signals through its heterodimeric receptor, composed of the specific IL-21 receptor (IL-21R) and the common gamma chain, and engagement of IL-21 with the IL-21R results in a signaling event primarily mediated by JAK/STAT-3. This cytokine plays an important role in T cell-dependent B cell responses by stimulating IgG production and promoting differentiation of activated B cells into plasma cells and memory cells within germinal centers (GC) [3–5]. IL-21 promotes GC B cell responses by both direct signaling to B cells and by driving Tfh cell development and effector function [6]. In addition to its role in T-dependent B cell activation, IL-21R signals are also critical to maintain survival and prevent exhaustion of CD8^+^ T cells responding to chronic virus infection [7–9]. Furthermore, IL-21 promotes expression of RORγt and differentiation of Th17 and Tc17 cells [10,11]. These profound effects of IL-21/IL-21R signaling on B cell and T cell immune responses in other experimental systems suggested the possibility that IL-21R signaling could be important in host defense to IAV infection.

Gamma delta (γδ) T cells are innate-like T cells that express a TCR of limited diversity composed of γ and δ subunits (in contrast to conventional α and β subunits). γδ T cells are preferentially located at mucosal sites where they are thought to rapidly respond to pathogens and host-derived danger or stress signals [12]. In the context of IAV infection, pulmonary γδ T cells have been demonstrated to expand in the lung after IAV infection, and they contribute to the IL-17 response in lethal IAV infection [13]. Furthermore, drug-induced expansion of γδ T cells was shown to contribute to virus control[14]. Human γδ T cells express the IL-21R, and IL-21/IL-21R signaling has been demonstrated to influence the differentiation of a subset of γδ T cells with B cell-helping capabilities [15]. However, the role of IL-21/IL-21R signaling in regulating differentiation and/or function of γδ T cells in vivo has not been evaluated.

In this report we evaluated the contributions of IL-21/IL-21R signaling to immune responses in a mouse model of primary IAV infection using IL-21R KO mice. We found that lack of IL-21R signaling had no significant impact on virus clearance, adaptive T cell responses, or inflammatory myeloid cell accumulations in the lung. However, a subset of inflammatory cytokines, notably IL-17, was elevated in the bronchoalveolar lavage fluid of IL-21R KO mice, corresponding with a small increase in morbidity (as measured by weight loss). Furthermore, we observed that there was a large increase in respiratory γδ T cells capable of producing IL-17 in IL-21R KO mice after IAV infection and at steady state. Finally, we found that IL-21R signaling suppressed IL-17-producing γδ T cells intrinsically. The implications of IL-21R signaling in IAV infection and IL-17^+^ γδ T cell function are discussed.

## Results

### IL-21R deficiency has a minimal impact on adaptive immune responses and virus clearance during primary IAV infection

**Fig 1:**
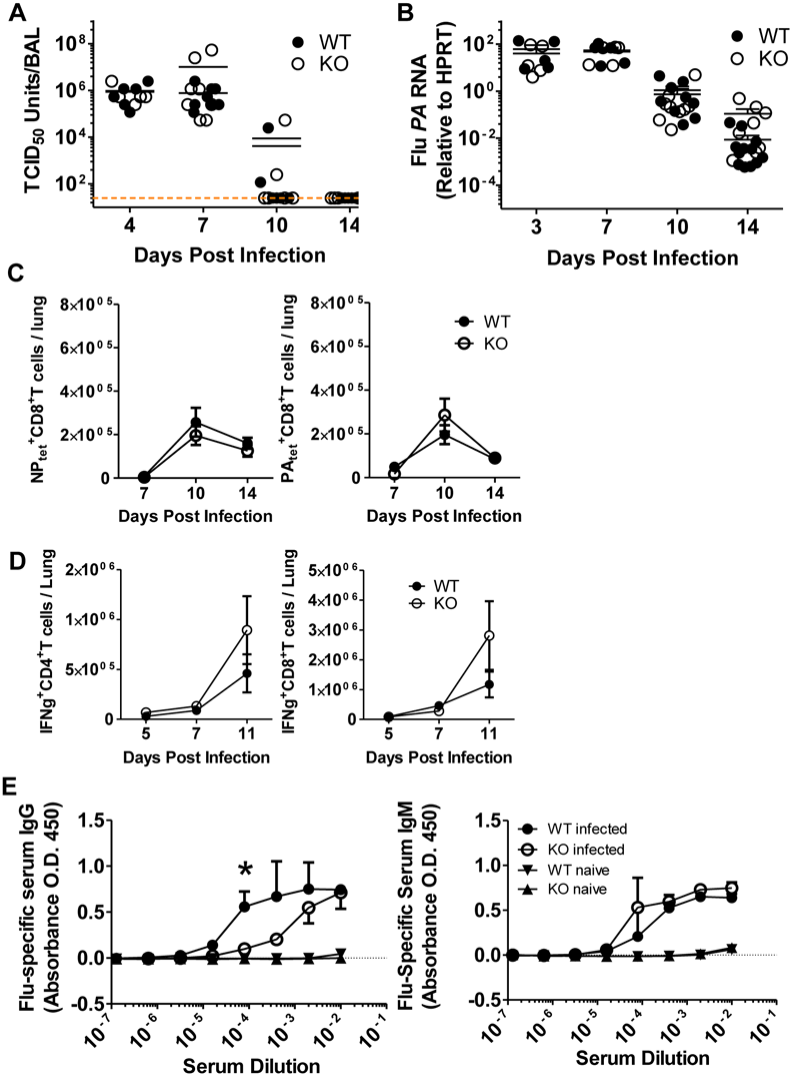
IL-21R deficiency has a minimal impact on adaptive immune responses and virus clearance during primary IAV infection. WT C57BL/6 mice or IL-21R KO mice were infected with 0.1LD_50_ PR8 and BAL, lung homogenate, lung cell suspensions, or serum was collected at various days post-infection as indicated. (A) Infectious virions in the BAL were quantified by TCID_50_ analysis (n = 5–8, combined from 3 independent experiments, dashed line represents threshold of detection). (B) Expression of the IAV *PA* gene in RNA from whole lung homogenate was measured by qRT-PCR (day 3–5: n = 5, day 7–10: n = 9–12, combined from 2 or more independent experiments). (C) CD8^+^ T cells specific for the IAV NP_366_ and PA_244_ epitopes were identified in lung cell suspensions by tetramer staining and flow cytometry (n>6, combined from 2 or more independent experiments). (D) Lung cell suspensions were stimulated ex vivo with PMA/Ionomycin for 4 hours and IFNγ^+^ CD4^+^ and CD8^+^ T cells were quantified by intracellular cytokine staining and flow cytometry (n = 4, combined from 2 independent experiments). (E) IAV specific serum IgG and IgM antibody levels were quantified by ELISA (n = 3–4).

IL-21/IL-21R signaling has been demonstrated to play a critical role in both T and B lymphocyte function by preventing exhaustion of antiviral CD8^+^ T cells in chronic infections [7–9] and promoting the differentiation of germinal center B cells leading to generation of high affinity class-switched antibodies [3,5,16]. Since optimal clearance of IAV depends on both antibody-mediated virus neutralization [1] and killing of influenza-infected cells by CD8^+^ T cells [2], we wanted to investigate what role, if any, IL-21R signaling played in a mouse model of IAV infection. To this end, we intra-nasally infected IL-21R KO or wild type (WT) mice with a sub-lethal dose of Influenza A/PR/8/34 (PR8) and measured viral loads at various times post infection. We found that clearance of infectious virus from the bronchoalveolar lavage (BAL) fluid was comparable in IL-21R KO mice and WT mice as measured by TCID_50_ assay (Fig 1A). Normal virus clearance in IL-21R KO mice was also evident in the similar levels of the viral polymerase gene (PA) detected in the infected lung (Fig 1B).

Although virus was cleared with similar kinetics, we wanted to determine if adaptive immune responses in IL-21R KO mice were altered. To this end, we evaluated antigen specific CD8^+^ T cell responses in IL-21R KO mice at various days post infection by employing fluorescently labeled tetramers that recognize TCRs specific for the IAV immunodominant epitopes, NP_366_ or PA_244_, presented by MHC class I molecules. We found no change in the total numbers of antigen specific CD8^+^ T cells in the lungs following infection (Fig 1C). Furthermore, when we re-stimulated lung cell suspensions with PMA/ionomycin ex vivo, we found no difference in the quantity of IFNγ producing CD4^+^ or CD8^+^ T cells (Fig 1D). Although there was a trend toward increased IFNγ^+^ T cells in IL-21R KO mice at day 11 p.i., this did not reach statistical significance.

Next, we evaluated IAV-specific antibody responses in IL-21R KO mice, since IL-21/IL-21R signaling on B cells is required for optimal germinal center-derived high affinity class-switched antibody production. To this end, we measured IAV-specific IgG and IgM in sera collected from infected IL-21R KO and WT mice at day 10 post-infection. Interestingly, we found a reduction in IAV-specific IgG, but not IgM (Fig 1E), consistent with the important role of IL-21R signaling on antibody class switching [3,5]. However, it appeared that the reduced IgG response in IL-21R KO mice on day 10 p.i. did not impair their ability to clear virus during primary IAV infection (Fig 1A). This finding is in support of the view that IgM, not IgG, is the major antibody class responsible for clearance of primary IAV infection [17,18]. Taken together, these data demonstrate that IL-21R signaling is not required for the development of adaptive immune responses and normal virus clearance after primary IAV infection.

### IL-21R signaling suppresses IL-17-associated cytokine production in the lung after IAV infection

**Fig 2:**
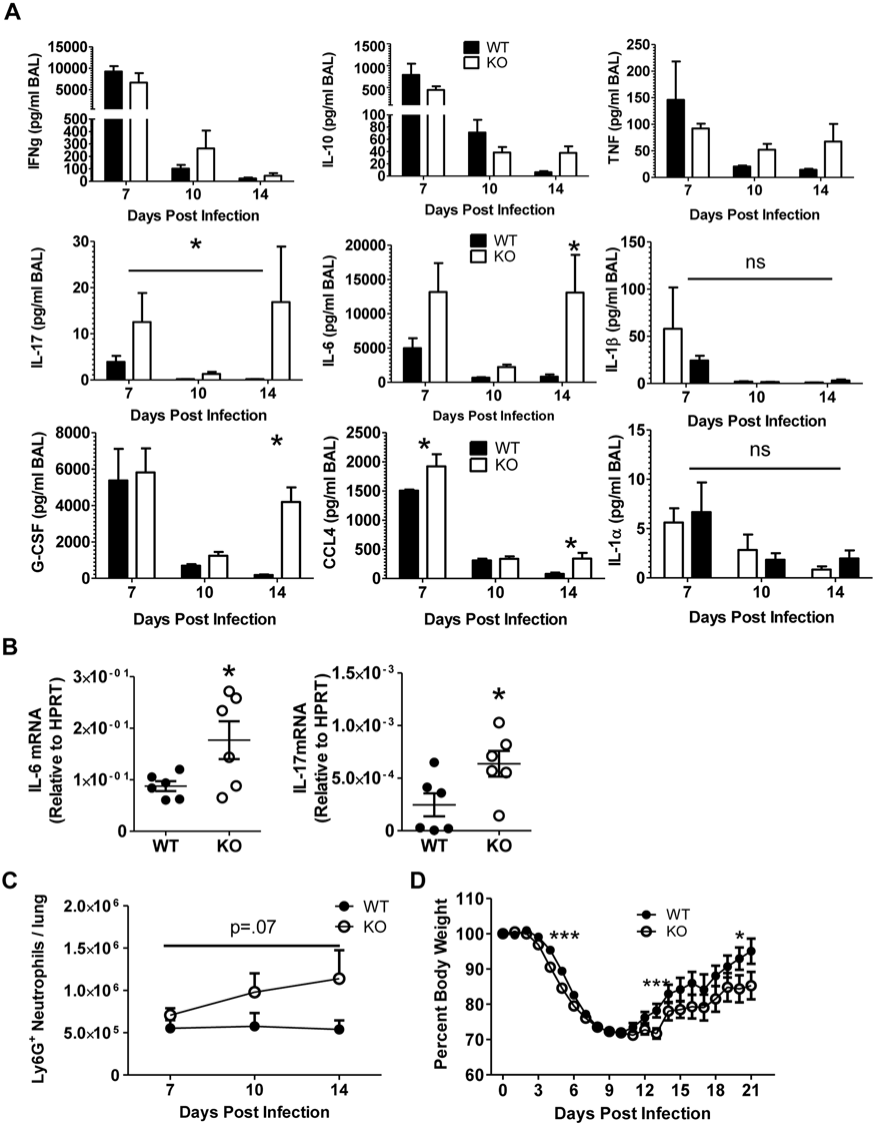
IL-21R signaling suppresses IL-17-associated cytokine production in the lung after IAV infection. WT C57BL/6 mice or IL-21R KO mice were infected with 0.1LD_50_ PR8 and BAL, lung homogenate, or lung cell suspension was collected at various days post-infection as indicated. (A) BAL cytokines were analyzed by Luminex 30-plex cytokine array (n = 4–6, combined from 2 independent experiments). (B) Whole lung homogenates from animals on day 10 p.i. were analyzed for *IL6* and *IL17* gene expression by qRT-PCR (n = 6, combined from 2 independent experiments). (C) Neutrophils were quantified from lung cell suspensions (day 7: n = 3; day 10–14: n = 6–12, combined from 2 or more independent experiments). Neutrophils were CD11b^+^Ly6G^+^ SiglecF^-^. (A-C) * denotes p<0.05, ns = not significant.

In addition to evaluating IAV-induced adaptive immune responses, we wanted to probe potential IL-21R-dependent changes in the production of soluble mediators that could influence the inflammatory responses in the respiratory tract. To this end, we evaluated cytokine/chemokine production in the BAL fluid prepared from WT and IL-21R KO mice. We found that cytokines typically of effector T cell origin (e.g. IFNγ, IL-10, and TNF) were not significantly altered in WT and KO mice (Fig 2A), although there was a general trend toward higher cytokine levels in IL-21R KO mice late after infection (i.e. day 14 p.i.). This finding is consistent with our finding that antiviral CD4^+^ and CD8^+^ T cell responses were largely unaffected in the IL-21R KO mice (Fig 1). In contrast, we noted an increase in a subset of Th17-associated cytokines in the BAL, specifically IL-17, IL-6, G-CSF, and CCL4, and elevated levels of these cytokines were sustained even after the majority of the IFNγ response had subsided, i.e. day 14 post infection (Fig 2A). It is worth noting that in sub-lethal IAV infections, IL-17 protein levels are typically undetectable in the lungs after day 7 p.i. [19]. Several cytokines have been demonstrated to promote IL-17^+^ γδ T cell development, including IL-1α, IL-1β, and IL-23. Importantly, IL-1α and IL-1β were not changed (Fig 2A) and IL-23 was not detected (data not shown) in the BAL of IL21R KO mice during IAV infection, suggesting that IL-21R signaling suppresses the accumulation of IL-17^+^ γδ T cells in the lung, independent of the cytokines already reported to regulate γδ T cells. In addition to elevated proteins secreted into the BAL fluid, we observed increased mRNA transcripts for both IL-17 and IL-6 (Fig 2B) in the lungs of IL-21R KO mice. This elevated IL-17 and IL-6 might have contributed to the trend toward increased neutrophil infiltration into the infected lungs in IL-21R KO mice, although this did not reach statistical significance (Fig 2C). Similarly, IL-21R KO mice exhibited slightly increased weight loss (Fig 2D), possibly due to elevated cytokines late after infection. Collectively, these findings suggested that IL-21R signaling played a suppressive role in the production of Th17-associated cytokines, but loss of IL-21R signaling did not lead to overt increases in neutrophil accumulation or a large increase in host morbidity.

### Pulmonary IL-17^+^ γδ T cells are elevated in IL-21R KO mice during IAV infection

**Fig 3:**
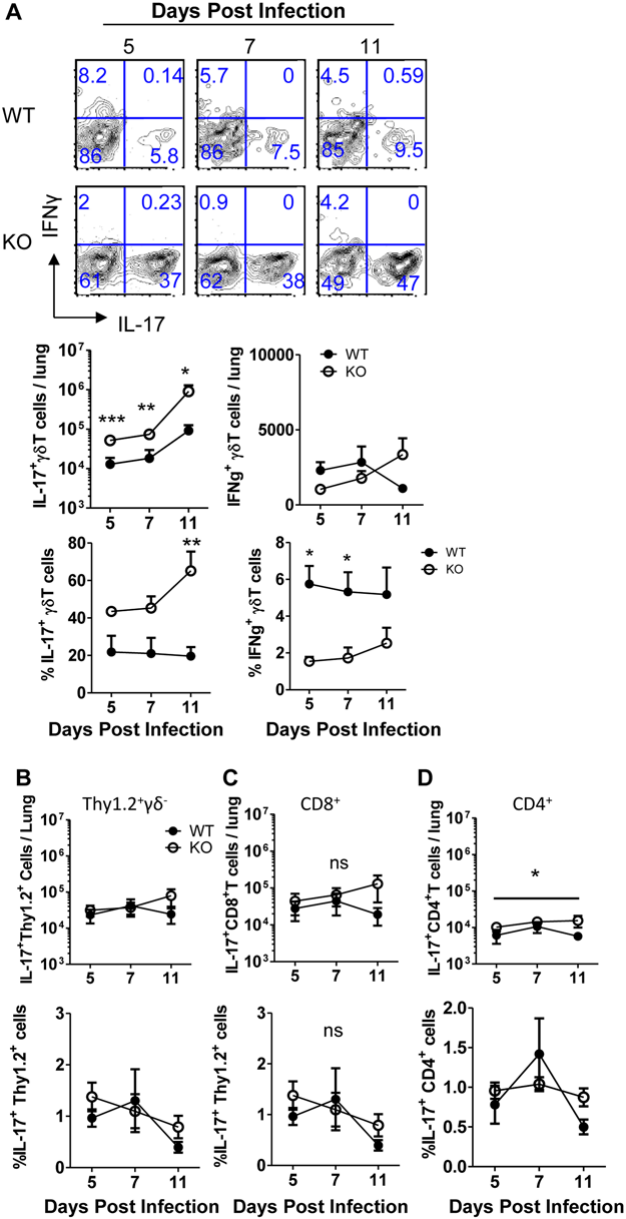
Pulmonary IL-17^+^ γδ T cells are elevated IL-21R KO mice during IAV infection. WT C57BL/6 mice or IL-21R KO mice were infected with 0.1 LD_50_ PR8 and lung cell suspensions were collected at various days post-infection as indicated. Cell suspensions were then stimulated with PMA/ionomycin for 4 hours in the presence of monensin, and IL-17 or IFNγ was measured by intracellular cytokine staining. (A) Cells were gated on Thy1.2^+^TCRδ^+^. Representative flow cytometry plots and quantification of frequencies and absolute numbers of cytokine positive γδ T cells are shown. (B-D) Quantification of frequencies and numbers of IL-17^+^ cells is shown. (B) total Thy1.2^+^TCRδ^-^, (C) CD8^+^, (D) CD4+. (A-D) n = 4, combined from 2 independent experiments.

IL-17 can be produced by a variety of lymphocytes, including CD4^+^ Th17 cells, CD8^+^ Tc17 cells, innate lymphoid cells (ILCs), and γδ T cells. Interestingly, IL-21 has been reported to be sufficient, though not required, to promote differentiation of Th17 cells in the presence of TGFβ [11], and CD8^+^ Tc17 cells develop in the presence of the same cytokine cues [10]. To identify the cellular source(s) of IL-17 that could account for the elevated IL-17 levels in the lungs of IAV infected IL-21R KO mice, total single cell lung suspensions were stimulated with PMA and ionomycin directly ex vivo to detect IL-17-producing lymphocytes. Since γδ T cells have also been previously documented to be capable of producing IL-17 during IAV infection [13], we analyzed IL-17-producing γδ T cells in the infected lungs of IL-21R KO mice at various times post infection. Unexpectedly, we observed a dramatic increase in the number and frequency of IL-17^+^ γδ T cells in the lungs of IL-21R KO mice compared to WT mice (Fig 3A). Interestingly, when we measured the capacity of TCRδ^+^ cells to produce either IFNγ or IL-17, we observed that a large fraction of IL-21R KO γδ T cells were IL-17^+^, but relatively few γδ T cells from either WT or IL-21R KO mice were able to produce IFNγ (Fig 3A). Furthermore, for the minority of γδ T cells that produced IFNγ upon stimulation, the absolute number of cells did not change in the IL-21R KO mice. Importantly, we saw no significant increase the total Thy1.2^+^ TCRδ^-^ population, which included CD4^+^ and CD8^+^ T cells, as well as ILCs (Fig 3B). Furthermore, there was no increase in CD8^+^IL-17^+^ T cells (Fig 3C). However, we saw a small but statistically significant increase in the number of IL-17^+^ CD4^+^ T cells, but these cells comprised such a small frequency of the total CD4^+^ T cell population that there was no corresponding increase in frequency of Th17 cells (Fig 3D).

Taken together, this data suggested that IL-21R signaling suppressed a subset of IL-17 producing γδ T cells in the respiratory tract, and in the absence of IL-21R these cells expand in number to encompass a larger proportion of the total γδ T cell pool.

### Intrinsic IL-21R signaling on γδ T cells suppresses the IL-17^+^ subset in the respiratory tract

**Fig 4:**
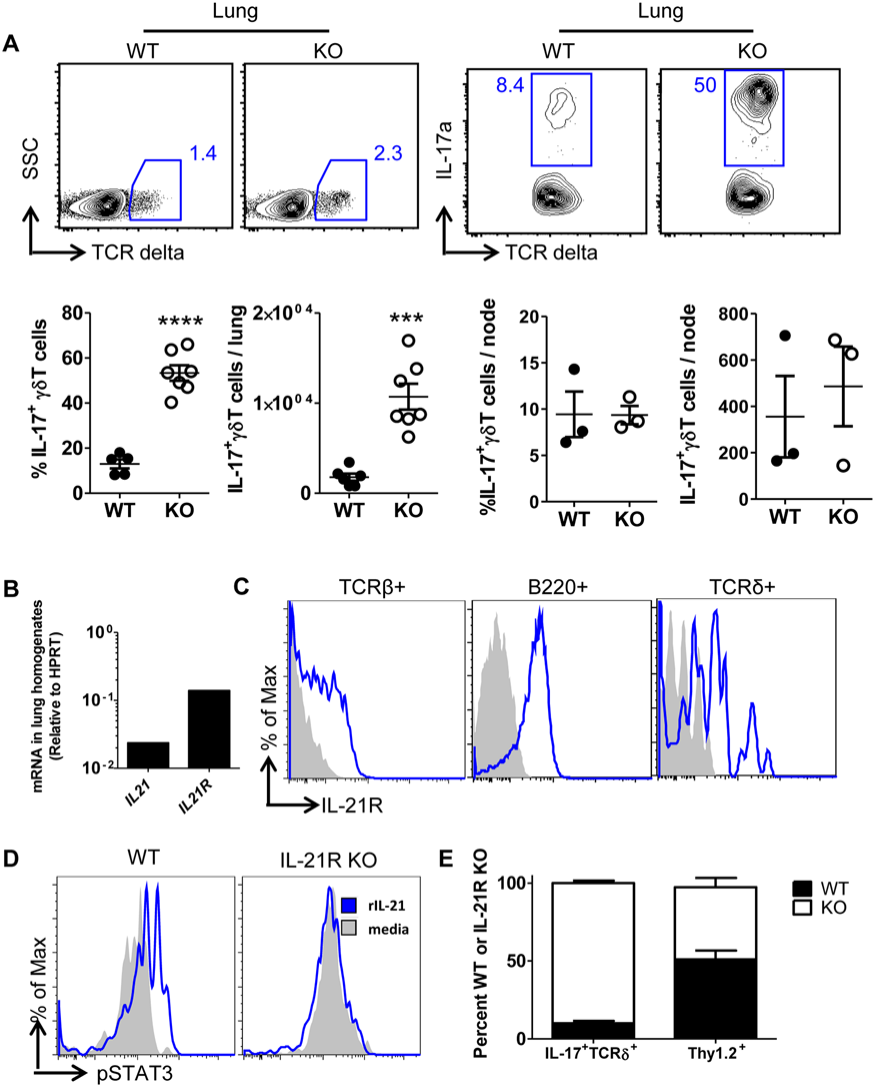
Intrinsic IL-21R signaling on γδ T cells suppresses IL-17^+^ subset in respiratory tract. Lung cell suspensions or whole lung homogenates from uninfected WT C57BL/6 and IL-21R KO mice were harvested, as indicated. (A) Cell suspensions were stimulated with PMA/ionomycin in the presence of monensin and IL-17^+^ γδ T cells were quantified by intracellular cytokine staining and flow cytometry. Representative flow plots and quantification of frequency and absolute numbers of lung IL-17^+^ γδ T cells are shown (Lung: n = 5–7, combined from 2 independent experiments, Node, n = 3) (B) Gene expression of *IL21* and *IL21R* from RNA of whole lung homogenate from WT mice. (C) IL-21R expression on the surface of lymphocytes in lung cell suspensions from WT mice was analyzed by flow cytometry. Solid gray line represents FMO control (representative of 2 independent experiments). (D) Lung cell suspensions from WT or IL-21R KO mice were treated with rIL-21 ex vivo for 15 minutes then cells were stained intracellularly for P-STAT3 (Blue = WT, red = IL-21R KO, gray = isotype control). (E) Lung cell suspensions were harvested from IL-21R KO/WT CD45.1 BM chimeric mice and stimulated ex vivo with PMA/ionomycin for 4 hours. IL-17 expression in CD45.1 WT or CD45.2 IL-21R KO cells was determined by intracellular cytokine staining and flow cytometry. Cells were gated on Thy1.2^+^ or TCRδ^+^IL-17^+^ and WT (CD45.1) and IL-21R KO (CD45.2) composition was determined within the population (n = 3, representative of 2 independent experiments).

Unlike CD4^+^ and CD8^+^ T cells which acquire their effector phenotypes upon activation in the periphery (e.g. draining lymph node), γδ T cells acquire their effector cytokine program in the fetal thymus [20,21]. We wanted to determine if IL-21R exerted its suppressive effect on the IL-17^+^γδ T cell subset only in the conditions of IAV infection, or if this effect could be observed at steady state, indicative of a potential role for IL-21R in the development/differentiation of the IL-17^+^γδ T cell subset. To this end we harvested lungs from uninfected WT and IL-21R KO mice, and identified IL-17^+^γδ T cells after ex vivo stimulation and intracellular cytokine staining. We observed that in the uninfected lung, but not the lung-draining lymph node, the frequency and numbers of IL-17^+^ γδ T cells were substantially increased in IL-21R KO mice, suggesting that IL-21R signaling was occurring independently of IAV infection to impact the γδ T cell effector phenotype in the lung (Fig 4A). This data suggested that IL-21 could engage IL-21R expressed by γδ T cells in the naïve lung.

To examine this possibility we analyzed mRNA for the *IL21* and *IL21R* genes in uninfected whole lung homogenates. Indeed, we detected both signaling components at the gene expression level (Fig 4B). Furthermore, we detected the presence of IL-21R on γδ T cells in the naïve lungs, along with IL-21R^+^ TCRβ^+^ T cells and B-cells, which are known to express the IL-21R. (Fig 4C). Since IL-21/IL-21R signaling occurs primarily through STAT3 [22], we stained for intracellular P-STAT3 in cultured lung cell suspensions that had been treated with recombinant IL-21 for 15 minutes. We found that γδ T cells were capable of responding to IL-21 to phosphorylate STAT3 (Fig 4D). Finally, to determine if the IL-21R signal was required to intrinsically suppress IL-17 producing γδ T cells, we generated 1:1 WT/IL-21R KO mixed bone marrow chimeras by reconstituting lethally irradiated WT mice with congenically marked WT (CD45.1) and IL-21R KO (CD45.2) bone marrows. After eight weeks, we harvested uninfected lung cell suspensions and stimulated them ex vivo with PMA and ionomycin, then measured the IL-17 producing capacity of γδ T cells by intracellular cytokine staining. We found that the majority of IL-17^+^ γδ T cells were derived from IL-21R KO origin in the BM chimeric mice (Fig 4E), suggesting that IL-21R signaling intrinsically suppressed development and accumulation of IL-17^+^ γδ T cells.

## Discussion

In this report we employed IL-21R KO mice to examine the role of IL-21R signaling in controlling the host response to IAV infection. While lack of IL-21R led to a reduction of the IAV-specific IgG response, IL-21/IL-21R signaling was not required for virus clearance or the induction and effector function of CD8^+^ T cell responses. Unexpectedly, however, we observed that IL-21R KO mice had increased levels of a subset of Th17-associated cytokines in the BAL, which was associated with a slight increase in weight loss and a dramatic increase in numbers of γδ T cells capable of producing IL-17. Furthermore, we demonstrated that IL-21R expressed on γδ T cells negatively regulated the accumulation of IL-17^+^ γδ T cells in the respiratory tract.

An important finding in this report is that in contrast to chronic viral infection models, IL-21R signaling is not required for acute antiviral immune responses and efficient virus clearance during primary IAV infection. Because of the well-documented importance of IL-21/IL-21R signaling in both CD8^+^ T cell responses and Tfh-dependent B-cell antibody production, we expected IL-21R KO mice to have impaired virus clearance after IAV infection. However, as we demonstrate in Fig 1, both infectious virions and viral genes are efficiently eliminated from the respiratory tract in IL-21R KO mice. This result can perhaps be attributed to the normal IgM responses in early time points prior to the evolution of high affinity anti-IAV IgG responses. Furthermore, normal virus clearance could be augmented by the enhanced innate immune responses mediated in part by IL-17-associated cytokines produced by γδ T cells. However, it is important to note that while we have shown that IL-21R signaling was not required for clearance of primary IAV infection, IL-21R signaling has been shown to play a critical role in the generation of CD8^+^ T cell and B cell memory during viral infection, thus likely plays an important role in pathogenesis and virus clearance after secondary IAV infection [4,23].

Cytotoxic CD8^+^ T cells are important for virus clearance because they have the ability to recognize and eliminate virally infected cells through interactions with viral peptide-loaded MHC Class I molecules, as evidenced by studies of IAV infection in mice deficient in MHC Class I [2]. In a chronic virus infection model (e.g. LCMV clone 13) where virus persists over 35 days, IL-21R KO CD8^+^ T cells exhibited a more prominent exhausted phenotype compared to WT mice, and viral loads were correspondingly higher. Under the conditions requiring prolonged effector activity, virus-specific CD8^+^ T cells may require IL-21R signaling to prolong their survival and prevent exhaustion [7]. However, IAV infection occurs acutely and its clearance is achieved in a relatively short time period (i.e. by day 10 p.i.). Thus, this short time period of virus infection may bypass any need for additional survival signals (i.e. IL-21R) to prolong CD8^+^ T cell function. The latter is consistent with the findings of studies using the acute LCMV Armstrong strain, in which the CD8^+^ T cell response was intact in IL-21R KO mice.

In addition to CD8^+^ T cell responses, IAV-specific antibody responses are required to achieve efficient virus clearance, as demonstrated by enhanced susceptibility to infection in B-cell deficient mice [1]. It is well demonstrated that while IL-21R signaling is not required for activation of short-lived antibody secreting cells, IL-21/IL-21R signaling plays a critical role in germinal center maintenance and generation of long-lived high affinity class-switched antibodies, which are responsible for providing long-term sterilizing immunity after virus infections [24]. In IAV infected mice, the GC-derived high affinity IgG response begins to be detectable on day 7 after infection but does not reach peak levels until 30 days post infection [25]. Since IAV infection is cleared from the respiratory tract by day 10 p.i., it is likely that antibody derived from germinal centers is not critically required for efficient virus clearance during primary infection. Rather, early production of IgM plays an important role in protecting the host from IAV infection [17]. In support of this, IL-21R deficient mice exhibit normal, if not elevated, levels of anti-IAV IgM. This robust IgM antibody is likely derived from extra follicular (non-germinal center) plasmablasts that are largely responsible for neutralization of virus during primary infection [26,27].

An unexpected and novel finding in this study was that lack of IL-21R resulted in a dramatic increase in IL-17^+^γδ T cells in the respiratory tract during IAV infection. IL-17^+^ γδ T cells are a subset of γδ T cells that preferentially reside in mucosal environments, and they have been most well studied as effectors of mucosal barrier function and participants in autoimmune diseases [28]. In the context of IAV infection, γδ T cells have been demonstrated to accumulate in the respiratory tract after infection, to contribute to IAV clearance after ex-vivo expansion in a humanized mouse model, and have been associated with regulation of late-stage inflammation [29]; however, a definitive functional role for endogenous γδ T cells in vivo during IAV infection has not been demonstrated [14]. We observed that in the absence of IL-21R, the number and frequency of IL-17^+^ γδ T cells were increased in the respiratory tract at steady state, and these cells expanded and accumulated in the lungs after IAV infection to much higher levels than in WT mice. Despite this increase in numbers, IL-17 cytokine levels in the BAL fluid were very low, although they were detectable late after infection, whereas virtually no IL-17 is detectable after day 7 in WT mice. These data suggest that γδ T cells did not spontaneously produce large quantities of IL-17 in the IAV infected respiratory tract, perhaps due to the strong Th1 response, which could antagonize the IL-17 response [30]. However, in a different disease model such as murine psoriasiform plaque formation, in which IL-17 and/or IL-17^+^ γδ T cells play an important role in disease progression [31], IL-21/IL-21R signaling deficiency could lead to a dramatic alteration in disease pathogenesis and provide a new system to study the role of IL-17^+^ γδ T cells.

Interestingly, the role of IL-21 / IL-21R signaling in immunity to pathogens has largely been described to promote αβ T cell and B cell function. In contrast, the characteristics and role of the γδ T cell compartment in contributing to disease pathogenesis in these studies has not been reported. Notably, recent studies have uncovered mutations in the *IL21R* gene in patients experiencing chronic intestinal cryptosporidium infections, correlating with decreased function of peripheral lymphocytes [32]. In light of these findings in IL-21R deficient humans, it would be interesting to determine if these patients also exhibit alterations in the quality and/or quantity of peripheral γδ T cells, especially in the gastrointestinal tract. In murine studies of *E*. *vermiformis* infection (a member of the Eimeriorina suborder of protozoan parasites related to cryptosporidium), γδ T cell deficiency alters disease progression, correlating with increased αβ T cell-mediated immune pathology [33]; thus, a possible change in the γδ T cell compartment of humans lacking functional IL-21R signaling could lead to changes in αβ T cell function in vivo as well as altered susceptibility to intestinal parasites like cryptosporidium.

Interestingly, in addition to slightly elevated IL-17 in the BAL fluid, we detected substantial increases in the cytokines IL-6, G-CSF, and CCL4, all of which can be induced by IL-17/IL-17R signaling in various cell types. IL-17 can stimulate both IL-6 and G-CSF production by airway epithelial cells [34], and IL-17 can stimulate CCL4 production by inflammatory macrophages [35]. Importantly, these mediators participate in recruitment or activation of neutrophils in the respiratory tract [34,36], contributing to IL-17 mediated protection from bacterial infection [37,38]. However, elevated levels of these cytokines in IL-21R KO mice did not translate into increased neutrophil accumulations in the respiratory tract during IAV infection. There was an apparent increase in neutrophils in the lung (Fig 2C), but this did not reach statistical significance, perhaps because the increase in BAL IL-17 was so small. Another potential explanation as to why the increased inflammatory cytokines did not lead to more cellular infiltration is that other regulatory mechanisms are at play during IAV infection that can limit neutrophil accumulation independently of IL-17 production, including regulatory T cells (manuscript under review). The evidence presented in this study suggests that although IL-17^+^ γδ T cells expanded and accumulated in the respiratory tract after IAV infection, they did not appear to have a major impact on pulmonary inflammation in the IAV infection system.

An important observation in this study was that IL-21R sends a signal directly on the γδ T cells to limit accumulation of the IL-17^+^ subset in the respiratory tract. Further studies would be needed to determine if IL-21R-mediated regulation of proliferation, survival, or migration controls the accumulation of γδ T cells in the lungs. It is notable that mRNA for IL-21 and the IL-21R are detectable in the lung at steady-state, suggesting that IL-21R engagement leading to suppression of IL-17^+^ γδ T cell accumulations could occur in the lung. Alternatively, since many γδ T cells acquire their effector program in the fetal thymus, IL-21/IL-21R signaling could play a pivotal role in the fate decision of these cells during development. According to the current understanding of γδ T cell differentiation, weak TCR signals received in the thymus lead to retained expression of SOX 13, up-regulation of RORγt, and differentiation to the IL-17^+^ phenotype, and away from the IFNγ^+^ subtype [21]. Therefore, IL-21R signaling could augment TCR signals and lead to a “strong” signaling event, favoring CD27^+^IL-17^-^ γδ T cell development; thus, in the absence of IL-21R signaling, γδ T cells preferentially become IL-17^+^. Future studies should evaluate IL-21/IL-21R signaling in development of IL-17^+^ γδ T cells.

Another interesting finding in this study was that IL-17^+^ γδ T cells but not IFNγ^+^ γδ T cells were increased in the lung, but not the lung draining lymph node (Fig 4A), suggesting that the role of IL-21R signaling in suppressing IL-17^+^ γδ T cells is both tissue- and cytokine-specific. Several distinct subsets of γδ T cells have been described based on their Vγ gene rearrangements, anatomical locations, and developmental origins. For example, IL-17 expressing γδ T cells expressing Vγ4 TCR are typically are found in the lung and secondary lymphoid organs, whereas IFNγ^+^ γδ T cells expressing Vγ7 and Vγ5 TCR are typically found in the gut, and skin, respectively[39]. Further investigation would be required to determine if other subsets in other tissues (e.g. skin and gut) are also controlled by IL-21R signaling.

Taken together, these studies describe the role of IL-21R signaling on adaptive and inflammatory immune responses during IAV infection. We demonstrate that IL-21R signaling is dispensable for control of primary IAV infection, and we point out a novel role for IL-21R in IL-17^+^ γδ T cell biology. These studies should be of interest to others investigating extra follicular IgM antibody responses in primary infections and γδ T cells in IL-17 dependent disease models.

## Materials and Methods

### Mice and Infections

C57BL/6 and CD45.1 mice were purchased from the National Cancer Institute (NCI). IL-21R KO mice were a kind gift from Dr. Warren Leonard, and were bred in-house. All mice were housed at the University of Virginia in a pathogen-free environment. Mice used in experiments were between 8–12 weeks old and matched for age and sex. Type A influenza virus A/PR/8/34 (H1N1) was grown in day 10 chicken embryo allantoic cavities as described previously [40]. Mice were infected with 300 egg infectious doses (EID_50_) of A/PR/8/34 i.n. (corresponding to a 0.1 LD_50_ dose) unless otherwise noted.

### Preparation of tissue and single-cell suspensions

Mice were sacrificed by cervical dislocation. Lungs were perfused through the right heart with 10 mL PBS to remove cells from the vasculature. To prepare single cell suspensions, lungs were minced and digested in media containing 183 U/mL collagenase D (Worthington) for 45 minutes at 37°C. Lung tissue was then disrupted through a steel screen, and red blood cells were lysed with ACK lysis buffer. Live cells were determined by trypan blue exclusion and counted with a hemocytometer. To prepare total lung RNA, lungs were processed with an electric homogenizer in 1mL TRIzol (Invitrogen) and stored at -80°C.

### qRT-PCR

RNA was extracted from TRIzol (Invitrogen)-homogenized samples, and cDNA was generated using Superscript III (Life Technologies) according to the manufacturers’ protocols. qPCR was performed on a Life Technologies StepOne instrument using SYBR Green (Life Technologies) according to manufacturer’s instructions. Relative gene expression is calculated by the following formula: 2^(ΔCt), where ΔCt = Ct(HPRT)—Ct(gene of interest). PCR primer sequences are as follows: Flu PA (Fw 5'-CGG TCC AAA TTC CTG CTGCTG A-3’and Rev 5'-CAT TGG GTT CCT TCC ATC CA-3'), HPRT (Fw 5’-CTC CGC CGG CTT CCT CCT CA-3’and Rev 5’-ACC TGG TTC ATC ATC GCT AAT C-3’), IL6 (Fw 5’-ACG GCC TTC CCT ACT TCA CA and Rev 5’-TCC AGA AGA CCA GAG GAA ATT TT-3’), and IL17 (Fw 5’-GGA CTC TCC ACC GCA ATG A-3’ and Rev 5’TCA GGC TCC CTC TTC AGG AC-3’).

### Antibodies for flow cytometry

The following mAbs were purchased from BD or eBioscience (unless otherwise stated), as conjugated to FITC, Alexa-488, PE, PE-Cy7, PerCP-Cy5.5, APC, Alexa Fluor 647, APC-Cy7, or Alexa Fluor 780: CD4 (GK1.5), CD4 (L3T4), CD8α (53–6.7), CD11b (M1/70), CD11c (HL3), CD45 (30-F11), CD90.2 (53–2.1), Ly6G (1A8), Ly6C (AL-21), B220 (RA36B2), TCRβ (H57-597), TCRδ (GL3), IL-17a (TC11-18H10), IL-17a (eBio17B7), CD45.1 (A20), CD45.2 (104), P-STAT3 (clone D3A7 from Cell Signaling Technology) and IFNγ (XMG1.2). Anti–mouse CD16/32 used for F_c_ receptor blocking was isolated and purified in the University of Virginia Hybridoma Core Facility.

### Flow cytometry analysis

Cells were suspended in buffer containing PBS, 2% FBS, 10mM EDTA, and 0.01% NaN_3_. Fc receptors were blocked with anti-mouse CD16/32, and then cells were incubated with specific monoclonal antibodies or fluorescence minus one controls for 30 minutes at 4°C. Where indicated, after surface staining, intracellular cytokine staining staining was performed using the Cytofix/Cytoperm and Perm/Wash buffers (BD). Flow cytometry was performed on FACS Canto flow cytometers (BD), and data were analyzed using FlowJo (Tree Star, Inc.).

### BAL fluid and cytokine determination

Bronchial alveolar lavage (BAL) fluids were harvested by cannulating the trachea and injecting, then withdrawing 0.5 mL PBS into the airways three times. Cells were removed by centrifugation and supernatants were stored at -80°C until analyzed. Cytokines were quantified by a multiplex Luminex assay (University of Virginia Flow Cytometry Core Facility).

### Virus titer

We used a tissue culture infectious dose 50 (TCID_50_) assay followed by a hemagglutination assay to quantify infectious virus in BAL fluid, as previously described [41]. In brief, we infected Madin-Darby canine kidney cells with 10-fold dilutions of BAL fluid from infected mice, then incubated the cultures for 3 days at 37°C. Supernatants were collected and mixed with a half volume of 1% chicken red blood cells (Charles River Spafas) in PBS, and TCID_50_ units were calculated from hemagglutination patterns.

### Irradiation and BM transfer

Mice were irradiated with 1050 rads and, within 24 hours, received an i.v. infusion of red blood cell-lysed bone marrow cells (1x10^6^ cells) from uninfected IL-21R KO and CD45.1 mice, mixed at a 1:1 ratio. IL-21R KO/CD45.1 bone marrow chimeras were allowed to reconstitute for 8 weeks before lungs were harvested.

### In vitro stimulation and intracellular cytokine staining

Lung cell suspensions were harvested from mice and stimulated with 100 ng/mL Phorbol 12-myristate 13-acetate (Sigma) and 1μg/ml ionomycin (Sigma) for 4–5 hours in the presence of GolgiStop (BD). After culture, cells were stained for surface markers then fixed and permeablized (BD Cytofix/Cytoperm) and stained for intracellular cytokines.

### Phospho-STAT3 Staining

Lung single-cell suspensions were harvested from mice and cultured in complete RPMI medium for 15 minutes in the presence of 100ng/ml recombinant IL-21 (eBiosciences). Cells were fixed and permeabilized by sequential incubation with 4% PFA for 10 minutes, 0.5% TritonX for 30 minutes, and 90% methanol for 30 minutes. Cells were then stained for surface and intracellular P-STAT3 or isotype in Perm/Wash buffer (BD).

### Statistics

Unless otherwise noted, a student T test was used to compare two treatment groups. Comparisons of two or more groups over time were analyzed with the two-way analysis of variance test followed by the Bonferroni post-test. These statistical analyses were performed using Prism5 software (for Windows; GraphPad Software, Inc.). Results are expressed as means ± SEM. Values of P <0.05 were considered statistically significant (*).

### Ethics Statement

All animal experiments were conducted in accordance with the Animal Welfare Act (Public Law 91–579) and the recommendations in the Guide for the Care and Use of Laboratory Animals of the National Institutes of Health (OLAW/NIH, 2002). All animal experiments were carried out in accordance with protocols approved by the University of Virginia Animal Care and Use Committee (Protocol Number 2230).

For anesthesia, a mixture of Ketamine(20mg/ml)/Xylazine (2mg/ml) was injected intraperitoneally. Mice were euthanized by cervical dislocation.
